# Enhancing rapeseed germination by nano zinc oxide and zinc sulfate particles under interrupted irrigation

**DOI:** 10.1038/s41598-025-31616-8

**Published:** 2025-12-25

**Authors:** Seyed Davood Alavifard, Mansour Taghvaei, Ruhollah Naderi, Mohsen Edalat, Bahram Heidari, Beata Dedicova

**Affiliations:** 1 Department of Plant Production and Genetics, School of Agriculture, Shiraz University, Shiraz, Iran; 2 Department of Plant Breeding, Swedish University of Agricultural Sciences (SLU), Alnarp, Sweden

**Keywords:** *Brassica napus*, Hybrid, Nanoparticles, Treatment, Germination, Physiology, Plant sciences

## Abstract

The manuscript investigates the effect of zinc oxide nanoparticles and zinc sulfate (applied singly or in combination) on seed germination traits of four spring rapeseed cultivars, including three open-pollinated varieties (Delgan, Zafar, and RGS003) and the hybrid variety Hayola 50, under varying drought stress conditions imposed on the mother plants. The study employs a split–plot design over two years, assessing several germination and vigor indices. The results showed that application of zinc fertilizer in the form of a combination of foliar application of zinc sulfate and zinc oxide nanoparticles (at a concentration of 5 ppm) to the mother plant at three growth stages, namely germination, flowering, and pod formation, generally improves germination performance, especially under drought stress.

## Introduction

The mustard family comprises many economically important species that are widely utilized as oil and food sources, as well as for ornamental purposes. Rapeseed (*Brassica napus* L.) is commonly used as an oil crop^1^. One of the significant differences between rapeseed varieties in current agriculture is whether they are open-pollinated or hybrid. Compared with open-pollinated varieties, hybrid rapeseed varieties typically have larger seeds and maintain higher yields at optimal seed rates^2^.

Seed viability and early seedling vigor depend on environmental conditions during the mother plant’s growth stages, and reduced seed quality leads to poor germination and decreased seedling vigor, especially under stress. Seed germination, seedling emergence, and plant establishment are crucial aspects of rapeseed production and serve as key indicators of seed or seedling vigor. Seed vigor is typically highest during physiological maturity in most crop species. Another indicator of seed quality is the germination rate of the varieties. Varieties that achieve higher germination percentages more quickly have better seed quality and greater seed vigor. The germination rate is a crucial agronomic seed characteristic, and its low level results in nonuniform plant density in the field^3^. Another determining factor of seed quality is the seed vigor index, which influences seed quality by affecting final germination percentage and seedling length. Seeds with greater vigor can tolerate environmental stresses and, despite a high germination percentage, produce more vigorous seedlings^4^. Studies on the genetic diversity of drought tolerance during germination in rapeseed are limited. Such studies can lead to methods for rapid screening of varieties. Various indices are used to measure tolerance at this stage, some based on differences in emergence percentage or rate between two regions. In contrast, seedling growth and development characteristics can also be used to examine genotypes^5^. The assessment of germination traits and seed vigor in mother rapeseed plants subjected to end-of-season drought stress revealed that drought and heat stress resulted in the production of low-weight, wrinkled seeds, accompanied by a significant reduction in germination percentage and seedling shoot length^6^. Researchers have investigated the effects of drought stress and regular irrigation on the emergence and establishment of 10 spring rapeseed varieties in the field, reporting that drought stress applied to the mother plants reduced seed vigor^7^. Drought stress during plant growth, especially in the reproductive stage, primarily reduces plant yield and, ultimately, the viability of the resulting seeds. Seed weight is related to germination and seed vigor, such that seed vigor decreases with reduced seed weight due to stress^8^. Drought during seed formation and filling periods reduces germination^9^. Drought stress during the pollination and seed-filling stages causes pollen grain destruction. Still, it disrupts the production and transfer of photosynthetic materials to the seed, ultimately resulting in reduced seed weight. A slight reduction in germination indices, including germination percentage and rate, seed vigor, and seedling length, was reported in this study due to a slight decrease in seed weight and quality^10^. Drought stress during the seed formation and filling stages limits the transfer of materials to seeds. It shortens the seed-filling period, resulting in wrinkled seeds with low reserves, thereby significantly affecting seed vigor. Given that greater storage material in mother seeds leads to greater shoot and root growth and more potent, healthier seedlings, drought stress results in weaker seedlings due to reduced storage materials and seed quality in mother seeds^11^. Drought stress, nutrient application, and foliar spray timing had significant effects on germination percentage (GP), germination speed (GS), germination value (GV), and germination energy (GE) of the plants. The maximum GP, GS, GV, and GE were observed under the control condition (without drought stress), drought stress at 50% flowering, and treatment with Nanozinc + Nanoiron nutrients^12^. Poor seedling emergence or establishment is one of the most important factors contributing to reduced rapeseed yield. Earlier counting of germinated seeds can be suggested for the rapid evaluation of seed survival. Additionally, seedling emergence and establishment in these seeds need to be evaluated. MGT can be used as a seed vigor index in rapeseed^13^.

Zinc can act as a cofactor for peroxidase and superoxide dismutase enzymes. These are practical factors in plant defense mechanisms under drought stress that increase plant resistance to free radicals, such as hydroxyl radicals and peroxides. This element mitigates the adverse effects of drought by regulating stomatal and ionic balance in crops^14^. Compared with small-grain cereals such as wheat, rapeseed can absorb and remove more zinc from the soil, potentially up to twice as much^15^. The effectiveness of zinc fertilizers depends on particle size. A reduction in particle number increases the unit weight, specific surface area, and solubility of nano fertilizers, thereby increasing the surface area available for fertilizer absorption and, ultimately, improving yield and plant strength. The application of nanoscale zinc oxide positively affects the germination, growth, and seed vigor of peanuts^16^. In this context, the foliar application of nanoformulated micronutrients is more suitable than soil application due to its potential to rapidly correct deficiencies, ease of application, ability to minimize micronutrient toxicity from accumulation, and capacity to prevent trace element fixation in soil^17^. NPs interact with plants and, depending on their properties (as nanoparticle sensors), cause various morphological and physiological changes in plants. The impact of nanoparticles is determined by their chemical composition, size, surface coating, reactivity, and, most importantly, effective dose^18^. Due to their small size and high specific surface area, nanoparticles can be readily absorbed through root stomata. If their diameter is less than 20 nanometers, they can pass through plant cell walls without any barrier^19^. Zinc oxide nanoparticles are highly water-soluble, and plants can absorb and accumulate them in their biomass, marking a significant turning point in the agricultural industry^20^. NPs can be absorbed through roots and leaves, affecting growth, crop production, biological membrane integrity, and the development of plant organs and seeds^21^. Studies have shown that a high zinc content in seeds can increase seed survival and establishment, particularly in areas with zinc deficiency^22^. Seed quality, measured in terms of seed vigor and seed density indices, was highest in seeds obtained from plants treated with 200 mg/L iron nanoparticles and 300 mg/L zinc nanoparticles compared to other treatments in both environments. The increase in seed vigor index was due to a significant increase in seedling length and seedling dry weight^23^. Information on seed quality and physico-biochemical changes that occur during germination in improved seeds from plants treated with nanoparticles is scarce^24^.

Introducing new technologies in crop production and examining their ability to overcome stresses can provide a new horizon for increasing plant production. Therefore, this study aimed to evaluate the potential of newly synthesized ZnO nanoparticles to enhance rapeseed seed germination under drought stress. Additionally, the effects of drought stress on specific seed germination parameters and the physiological characteristics of rapeseed seeds produced from mother plants exposed to drought stress, as well as their subsequent seedlings, were investigated.

## Materials and methods

### Experimental details

This experiment was conducted as a split-split plot design with a randomized complete block design and three replications in an agricultural field in Khuzestan Province, Behbahan County, over two years. The study site was located at 50° 10’ 42.866" N and 30° 44’ 19.629" E, at an elevation of 329 meters above sea level.

### Soil analysis

Soil samples were collected from 0 to 30 cm at the test site before planting the crop and tested. Soil analysis showed that the tested soil was a silty loam. Soil samples were analyzed to determine the physicochemical properties and zinc status of the test site according to standard methods (Table 1).

**Table 1:** Chemical and physical properties of the soil of the field.

***Texture***				***PH***	***EC*** (ds/m)	***OC*** (%)	***N*** (%)	***P*** (ppm)	***K*** (ppm)	***Zn*** (ppm)	***Fe*** (ppm)	***Cu*** (ppm)	***Mn*** (ppm)
***Sand***%	***Silt***%	***Clay***%	***TNV*** %										
20	75	5	11.55	7.61	1.8	0.91	0.07	11	280	0.92	5.5	1.01	6.3
*Silty loam*													

TVN is total neutralizing value, PH is the potential of hydrogen ion, Ec is electrical conductivity, Oc is organic carbon, N is nitrogen, P is phosphorus, K is potassium, Zn is zinc, Fe is iron, Cu is copper, and Mn is manganese.

### Experimental treatments and design

The experimental factors included three levels of irrigation interruption stress as follows: 1) control 2) water cut-off at the flowering stage 3) water cut-off at the pod formation stage as the main plot and four levels of zinc fertilizer combinations as follows: 1) no use of zinc (control) 2) zinc sulfate applied to the soil (20 kg/ha) 3) foliar application of Nano-zinc oxide 4) zinc sulfate applied to the soil + foliar application of Nano-zinc oxide as a sub-plot and four spring rapeseed cultivars (Delgan, Zafar, RGS003 and hybrid (Hyola50) were considered as sub-sub-plot. The design consisted of a central plot (4 × 72 = 288 m^2^), a subplot (4 × 18 = 72 m^2^), and a sub-subplot (3 × 6 = 18 m^2^), and was implemented in three replications.

### Cultivation practices

**Fig 1:**
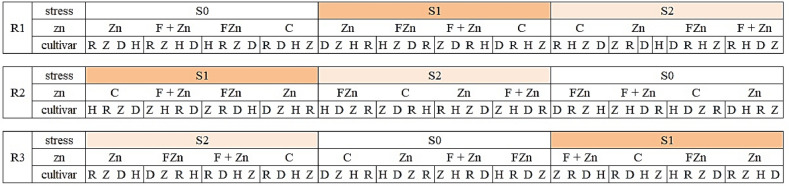
Schematic diagram of the experimental design (split split plot).

**Fig 2:**
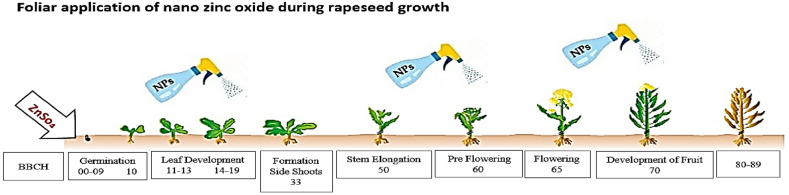
Schematic representation of the experimental design for foliar application of nanozinc oxide in rapeseed growth stages.

The planting steps were as follows: in each plot, 7 planting lines were planted with a row spacing of 40 cm. Small ridges were formed, then the surface of the ridges was smoothed, and a narrow groove was made on the ridges. In the plots where zinc sulfate was applied to the soil, fertilizer was applied as a strip in the grooves. Then, the seeds were evenly poured into the grooves, with an average distance of 2.5 cm and a depth of 2 cm, to achieve a density of 100 plants per square meter. They were then covered with soft soil. After sowing the seeds, irrigation was immediately carried out. During the growing season, three stages of foliar spraying of Nano zinc oxide with a dose of 5 ppm were carried out at three stages of rapeseed growth based on the BBCH scale as follows: the beginning of seedling growth (19 days after planting), the beginning of flowering (61 days after planting) and the beginning of pod formation (71 days after planting)^25,26^. Foliar spraying was carried out by a sprayer and by manual labor in the early morning to prevent evaporation and wind effects. In addition to the experimental treatments, urea fertilizer was applied in two stages (at the beginning of stem formation and at the beginning of flowering). To control broadleaf and narrowleaf weeds, post-emergence herbicides Lontrel (0.8 L/ha) and Galant Super (1 L/ha) were used, respectively. At the end of the growing season and after harvesting the plot, the necessary measurements were taken to determine the germination traits of the seeds obtained from the treatments (Figures 1 and 2).

### Measurement and calculation of germination-related indices

One hundred seeds from each treatment were placed on filter paper (BP) in Petri dishes, according to ISTA standards, and moistened with distilled water to examine the effects of the various treatments on the seeds. The germination percentage of germinated seeds in each Petri dish after seven days was considered the percentage. Normal and abnormal seedlings were identified and counted according to the International Seed Testing Association (ISTA) guidelines from days 5 to 10. On the final day of the germination tests, shoot and root lengths were measured with a ruler to millimeter precision. Finally, the average weight and length of 10 normal seedlings were calculated for each experimental unit. Using these counts and measurements, the following indices were calculated: germination percentage (GP), peak value of seeds (Ni), mean germination time (MGT), germination rate (GR), mean daily germination (MDG), germination value (GV), germination energy (GE), seedling length vigor index (SLVI), plumule length (PL), root length (RL), and the allometric coefficient (AC) (Table 2).

**Table 2:** Calculation relations of studied indicators.

**Row**	**Parameters**	**Formula**	**Reference**
1	Germination Percentage	GP = (N × 100)/M	^27^
2	Mean Germination Time	MGT = Σ (Ni × Ti)/N	^28^
3	Germination Rate	GR = 1/MGT	^28^
4	Mean Daily Germination	MDG = GP/T	^29^
5	Germination value	GV = GP × MDG	^29^
6	Germination Energy	GE=(Ni/N)×100	^30^
7	Allometric Coefficient	AC = PL/RL	^31^
8	Seedling length Vigor Index	SLVI = GP × Mean(PL+RL)/100	^32^

GP is Germination Percentage (%), MGT is Mean Germination Time (day), GR is Germination Rate (day^−1^), MDG is Mean Daily Germination (%), GV is Germination Value (-), GE is Germination Energy (-), SLVI is Seedling length Vigor Index (-), PL is Plumule length (cm), RL is Root length (cm), AC is Allometric Coefficient (-), N is Total number of germinated seeds at the end of the experiment, Ni is Number of seeds at peak germination, M is Total number of planted seeds, T is Duration of germination period (day), Ti is Number of days after start of germination (day).

### Statistical analyses

The data were analyzed via ‘SAS’ statistical software v.9.3 (SAS Institute Inc., Cary, NC, USA) after ensuring the homogeneity of variance by using the Kolmogorov–Smirnov and Shapiro–Wilk test The means were compared via Duncan’s multiple range test (DMRT) in cases where the F test of the ANOVA table indicated a significant difference, at least at the p < 0.05 and p < 0.01 levels, and the graphs were created via Excel (Microsoft Inc., Chicago, IL, USA).

## Results

This study performed variance analysis, comparing indices with significant interactions at different levels. Other indices were compared without significant interactions and analyzed separately; mean comparisons of their main effects were then performed.

### Germination percentage (GP)

The results of the analysis of variance showed that the effects of zinc fertilizer levels and irrigation levels on germination percentage were significant at the 1% probability level, and the impact of genotype and year was significant at the 5% probability level (Table 3).

**Table 3:** Variance analysis of the effects of treatments on the traits GP, MGT, GR, MDG and GV.

**S.O.V**	**df**	**GP**	**MGT**	**GR**	**MDG**	**GV**
year	1	7.03125 *	0.00026201 ns	0.00001549 ns	0.78145835 *	29812.3432 *
rep(year)	4	0.74652778 ns	0.00118359 **	0.00006944 **	0.08302937 ns	3198.2874 ns
Irrigation levels	2	43.33680556 **	0.00053759 ns	0.00003103 ns	4.81523225 **	184817.6949 **
year × Irrigation levels	2	6.88541667 **	0.00062272 **	0.00003637 **	0.76534263 **	28872.9513 **
rep × Irrigation levels (year)	8	0.87673611	0.00015189	0.00000891	0.09746113	3742.5833
Zinc fertilizer levels	3	6.08680556 **	0.00040446 ns	0.00002366 ns	0.67617758 **	25989.046 **
year × Zinc fertilizer levels	3	1.92939815 ns	0.00003287 ns	0.00000198 ns	0.21469508 ns	8123.0688 ns
Irrigation levels × Zinc fertilizer levels	6	0.22569444 ns	0.00018922 ns	0.00001111 ns	0.02512737 ns	982.5598 ns
year × Irrigation levels × Zinc fertilizer levels	6	0.89467593 ns	0.00005785 ns	0.00000336 ns	0.09932619 ns	3836.192 ns
rep × Zinc fertilizer levels (year × Irrigation levels)	36	0.75462963	0.00020094	0.00001177	0.08385988	3233.9244
Rapeseed cultivars	3	3.2349537 *	0.00017234 ns	0.00001009 ns	0.35950057 *	13921.401 *
year × Rapeseed cultivars	3	2.70717593 *	0.0000386 ns	0.0000023 ns	0.30049757 *	11658.2743 *
Zinc fertilizer levels × Rapeseed cultivars	9	0.41087963 ns	0.00006378 ns	0.00000374 ns	0.04564671 ns	1765.7097 ns
year × Zinc fertilizer levels × Rapeseed cultivars	9	2.11149691 *	0.00013438 ns	0.00000788 ns	0.23449679 *	9046.075 *
Irrigation levels × Rapeseed cultivars	6	1.27662037 ns	0.00011458 ns	0.00000646 ns	0.14164648 ns	5489.5904 ns
year × Irrigation levels × Rapeseed cultivars	6	0.7974537 ns	0.00007326 ns	0.00000432 ns	0.08858707 ns	3447.7512 ns
Irrigation levels × Zinc fertilizer levels × Rapeseed cultivars	18	1.2025463 ns	0.00004473 ns	0.00000261 ns	0.13357644 ns	5156.3418 ns
year × Irrigation levels × Zinc fertilizer levels × Rapeseed cultivars	18	1.42399691 ns	0.0001767 ns	0.00001036 ns	0.15829009 ns	6087.2416 ns
error	144	0.8576389	0.00012887	0.00000755	0.09527053	3682.581
CV(%)		0.94	0.55	0.55	0.94	1.89

ns, *, **: Representing nonsignificant and significant effects at 5 and 1% probability level. S0 is No stress; S1 is Stress in flowering; S2 is Stress in pod formation. GP is Germination Percentag (%); MGT is Mean Germination Time (day); GR is Germination Rate (day^−1^); MDG is Mean Daily Germination (%); GV is Germination value (-).

**Fig 3:**
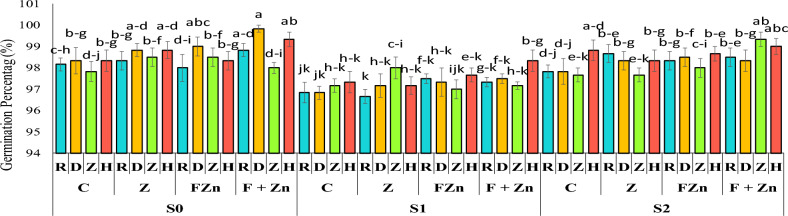
The combined effect of treatments on seed germination percentage in two years of experiment. Stress levels: S0 – No stress; S1 - Stress in flowering; S2 - Stress in pod formation. Zinc fertilizer levels: C – Control; Z - Zinc sulfate; FZN - Foliar zinc Nano oxide; F+ZN - Foliar zinc Nano oxide + Zinc sulfate. Canola genotypes: R – RGS003; D – Delgan; Z – Zafar; H- Hyola 50. Bars that have at least one letter in common are not significantly different by Duncan’s test at the 5% probability level.

The comparison of mean data showed that the germination percentage of first-year seeds (98.23%) was higher than that of second-year seeds (97.92%) (Table 4.). The highest seed germination percentage, 99.83%, was observed in the open-pollinated genotype Delgan following combined foliar applications of nano zinc oxide and zinc sulfate under normal irrigation conditions. After that, the hybrid genotype Hyola 50 had the highest seed germination percentage (99.33%) when treated with a combined foliar application of nano zinc oxide and zinc sulfate under normal irrigation conditions. Also, comparison of the mean data showed that the combined use of foliar application of nano zinc oxide and zinc sulfate was able to moderate the effect of drought stress on the percentage of seed germination, so that seeds from the Hyola 50 cultivar had a germination rate of 98.33% under severe drought stress conditions (water cut-off at the flowering stage) (Figure 3).

**Table 4:** Mean comparison test: effect of year on traits GP, MGT, GR, MDG and GV.

**Year**	**GP**	**MGT**	**GR**	**MDG**	**GV**
first year (2021)	98.2361 a	2.031835 a	0.4921854 a	32.74535 a	3217.187 a
second year (2022)	97.9236 b	2.033742 a	0.4917215 a	32.64117 b	3196.838 b

Numbers that have at least one letter in common in each column have no significant difference with Duncan’s test at the 5% level. GP is Germination Percentag (%); MGT is Mean Germination Time (day); GR is Germination Rate (day^−1^); MDG is Mean Daily Germination (%); GV is Germination value (-).

### Mean germination time (MGT)

The results of the variance analysis showed that the interaction between year and irrigation levels on the mean germination time was significant at the 1% level (Table 3).

**Fig 4:**
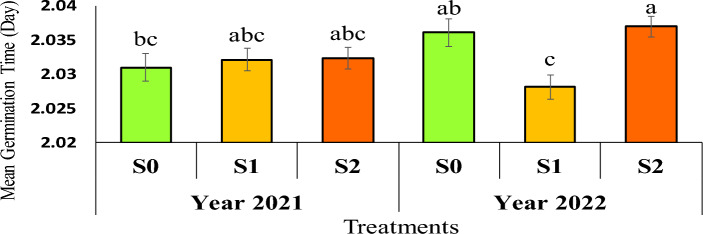
The reciprocal effect of year on irrigation levels on mean germination time. Stress levels: S0 – No stress; S1 - Stress in flowering; S2 - Stress in pod formation. Bars that have at least one letter in common are not significantly different by Duncan’s test at the 5% probability level.

The results of comparing the average data showed that the lowest average germination time in the second year was 2.028 days, and related to seeds obtained from the mother plant under severe drought stress conditions (water cut-off at the flowering stage), and the highest average germination time was 2.036 days, and related to seeds obtained from the mother plant under mild drought stress conditions (water cut-off at the pod formation stage) (Figure 4).

### Germination rate (GR)

The results of the variance analysis showed that the interaction between year and irrigation levels on germination rate was significant at the 1% level (Table 3).

**Fig 5:**
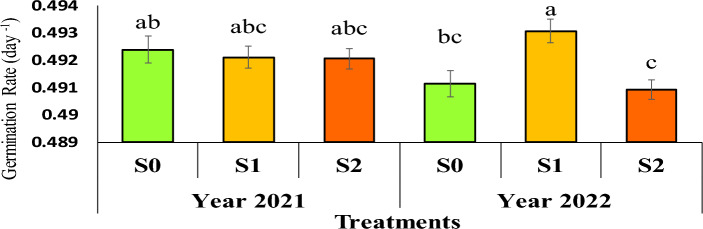
The reciprocal effect of year on irrigation levels on germination rate. Stress levels: S0 – No stress; S1 - Stress in flowering; S2 - Stress in pod formation. Bars that have at least one letter in common are not significantly different by Duncan’s test at the 5% probability level.

The average comparison showed that the highest germination rate occurred in the second year, with an average of 0.493 per day for seeds obtained from the mother plant under severe irrigation stress (water cut-off at the flowering stage) (Figure 5).

### Mean daily germination (MDG)

The analysis of variance showed that the effects of irrigation and zinc fertilizer levels on mean daily germination were significant at the 1% level, and the impact of genotype and year was significant at the 5% level (Table 3).

**Fig 6:**
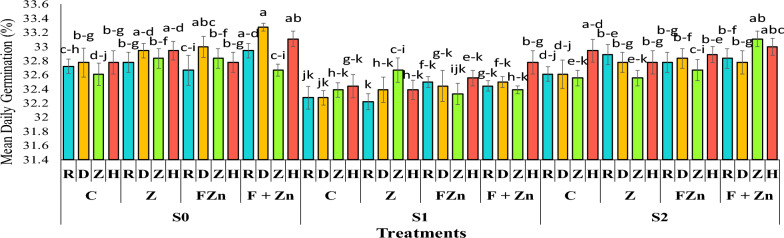
The combined effect of treatments on mean daily germination in two years of experiment. Stress levels: S0 – No stress; S1 - Stress in flowering; S2 - Stress in pod formation. Zinc fertilizer levels: C – Control; Z - Zinc sulfate; FZN - Foliar zinc Nano oxide; F+ZN - Foliar zinc Nano oxide + Zinc sulfate. Canola genotypes: R – RGS003; D – Delgan; Z – Zafar; H- Hyola 50. Bars that have at least one letter in common are not significantly different by Duncan’s test at the 5% probability level.

The comparison of mean data showed that first-year seeds had higher daily germination rates, averaging 32.74%, compared with second-year seeds, which averaged 32.64% (Table 4.). The highest mean daily germination was observed in open-pollinated Delgan genotype seeds, with an average of 33.27% in the treatment combining foliar applications of nano zinc oxide and zinc sulfate under normal irrigation conditions. After that, the hybrid genotype Hyola 50 showed the highest mean daily germination rate, averaging 32.11% in the treatment combining foliar applications of nano zinc oxide and zinc sulfate under normal irrigation conditions. Also, comparison of the mean data showed that the combined use of foliar application of nano zinc oxide and zinc sulfate was able to moderate the effect of drought stress on the mean daily germination of seeds, so that seeds from the Hyola 50 genotype had a germination rate of 32.77% under severe drought stress conditions (water cut-off at the flowering stage) (Figure 6).

### Germination value (GV)

The analysis of variance showed that the effects of irrigation levels and zinc fertilizer levels on germination value were significant at the 1% level. Additionally, the impacts of genotype and year on germination value were significant at the 1% probability level (Table 3).

**Fig 7:**
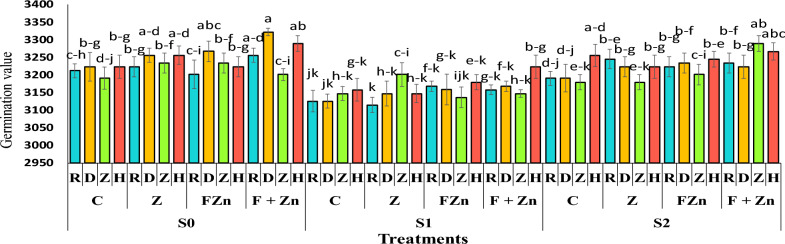
The combined effect of treatments on seed germination value in two years of experiment. Stress levels: S0 – No stress; S1 - Stress in flowering; S2 - Stress in pod formation. Zinc fertilizer levels: C – Control; Z - Zinc sulfate; FZN - Foliar zinc Nano oxide; F+ZN - Foliar zinc Nano oxide + Zinc sulfate. Canola genotypes: R – RGS003; D – Delgan; Z – Zafar; H- Hyola 50. Bars that have at least one letter in common are not significantly different by Duncan’s test at the 5% probability level.

The comparison of mean data showed that seed germination in the first year of the experiment was higher, with an average of 3217.18, than in the second year, with an average of 3196.83 (Table 4.). The highest germination value of 3322.3 belonged to the seeds of the open-pollinated Delgan genotype under the treatment of combined foliar application of nano zinc oxide and zinc sulfate under normal irrigation conditions. After that, the seeds of the hybrid genotype Hyola 50 ranked second, with a germination value of 3289.2, under the combined foliar application of nano zinc oxide and zinc sulfate, under normal irrigation conditions. Also, the comparison of the mean data showed that the use of zinc treatments was able to moderate the effect of drought stress on the germination value of seeds, so that the hybrid Hyola 50 and the open-pollinated genotype Delgan in the treatment of combined foliar application of nano zinc oxide and zinc sulfate and under severe drought stress conditions (water cut-off at the flowering stage) were able to achieve the highest germination value with 3223.6 and 3168.8, respectively. Still, under mild drought stress conditions (water cut-off at the pod formation stage), the cultivars Zafar and Hyola 50, in the treatment of combined foliar application of nano zinc oxide and zinc sulfate, had the highest germination values, with 3289.2 and 3267.2, respectively (Figure 7).

### Germination energy (GE)

The results of the mean-comparison showed that the interaction between year and irrigation levels on germination energy was significant at the 1% level (Table 5.).

**Table 5.** Variance analysis of the effects of treatments on the traits GE, SLVI, PL, RL and AC.

**S.O.V**	**df**	**GE**	**SLVI**	**PL**	**RL**	**AC**
year	1	2.60623475 ns	15.388765 **	0.91125 ns	7.41125 **	0.02152034 ns
rep(year)	4	11.84056967 **	1.3292522 **	0.36052083 **	0.39791667 **	0.00410421 **
Irrigation levels	2	5.37854117 ns	140.7127296 **	26.58760417 **	37.04 **	0.00665496 ns
year × Irrigation levels	2	6.24784098 **	4.026685 **	0.25260417 **	3.33291667 **	0.01423267 **
rep × Irrigation levels (year)	8	1.51737078	0.5720853	0.2684375	0.16296875	0.00641411
Zinc fertilizer levels	3	4.04849248 ns	47.1860959 **	7.23888889 **	15.79708333 **	0.00289252 ns
year × Zinc fertilizer levels	3	0.32816881 ns	2.7787122 **	0.22828704 **	1.5900463 **	0.00944277 **
Irrigation levels × Zinc fertilizer levels	6	1.8926251 ns	0.949577 ns	0.24552083 *	0.27013889 ns	0.00232377 ns
year × Irrigation levels × Zinc fertilizer levels	6	0.57750711 ns	0.465322 **	0.04811343 *	0.2612963 **	0.00195393 **
rep × Zinc fertilizer levels (year × Irrigation levels)	36	2.01068364	0.4316013	0.09292824	0.18892361	0.00153797
Rapeseed cultivars	3	1.73249252 ns	19.7740966 **	4.20787037 **	6.72023148 **	0.00025802 ns
year × Rapeseed cultivars	3	0.38471974 ns	0.3906637 **	0.06523148 *	0.13115741 **	0.00051363 ns
Zinc fertilizer levels × Rapeseed cultivars	9	0.63820407 ns	0.05017 ns	0.01237654 ns	0.05087963 ns	0.00085317 ns
year × Zinc fertilizer levels × Rapeseed cultivars	9	1.34381011 ns	0.0907026 ns	0.01609568 ns	0.01723765 ns	0.00022355 ns
Irrigation levels × Rapeseed cultivars	6	1.14494545 ns	0.2260913 **	0.03505787 ns	0.05884259 ns	0.00021287 ns
year × Irrigation levels × Rapeseed cultivars	6	0.73294435 ns	0.1849366 *	0.02478009 ns	0.06574074 *	0.00017972 ns
Irrigation levels × Zinc fertilizer levels × Rapeseed cultivars	18	0.44681802 ns	0.0865323 ns	0.00988812 ns	0.02953704 ns	0.0002465 ns
year × Irrigation levels × Zinc fertilizer levels × Rapeseed cultivars	18	1.76697854 ns	0.0470462 ns	0.02411651 ns	0.01677469 ns	0.00056338 ns
error	144	1.2885016	0.0688161	0.0179051	0.0299769	0.00049122
CV(%)		1.17	1.99	2.32	2.27	2.92

ns, *, **: Representing nonsignificant and significant effects at 5 and 1% probability level. S0 is No stress; S1 is Stress in flowering; S2 is Stress in pod formation. GE is Germination Energy (-); SLVI is Seedling length Vigor Index (-); PL is Plumule length (cm); RL is Root length (cm); AC is Allometric Coefficient (-).

**Fig 8:**
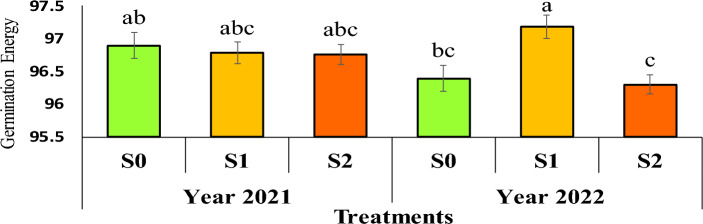
The reciprocal effect of year on irrigation levels on germination energy. Stress levels: S0 – No stress; S1 - Stress in flowering; S2 - Stress in pod formation. Bars that have at least one letter in common are not significantly different by Duncan’s test at the 5% probability level.

The results of the mean comparison showed that the highest germination energy in the second year, with a mean of 97.18, was associated with seeds from the mother plant under severe irrigation stress (water cut-off at the flowering stage) (Figure 8).

### Seedling length vigor index (SLVI)

The results of the variance analysis showed that the effects of year, irrigation levels, zinc fertilizer levels, genotype, and the interaction between irrigation levels and genotype on seedling length and vigor index were significant at the 1% probability level (Table 5.).

**Fig 9:**
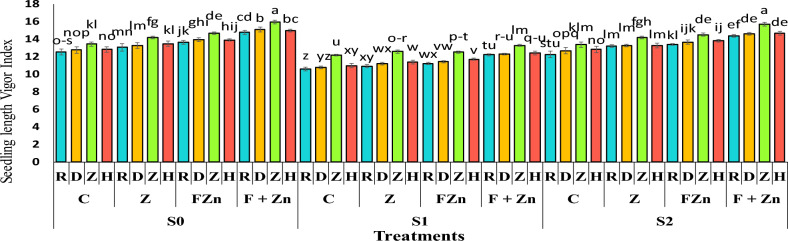
The combined effect of treatments on seedling length vigor index in two years of experiment. Stress levels: S0 – No stress; S1 - Stress in flowering; S2 - Stress in pod formation. Zinc fertilizer levels: C – Control; Z - Zinc sulfate; FZN - Foliar zinc Nano oxide; F+ZN - Foliar zinc Nano oxide + Zinc sulfate. Canola genotypes: R – RGS003; D – Delgan; Z – Zafar; H- Hyola 50. Bars that have at least one letter in common are not significantly different by Duncan’s test at the 5% probability level.

The results of the mean comparison showed that the seedling length vigor index in the first year (13.36) was higher than in the second year (12.9; Table 6.). The highest seedling length vigor index belonged to the Zafar genotype seeds with an index of 15.94 under the treatment of combined foliar application of nano zinc oxide and zinc sulfate under normal irrigation conditions. After that, the Delgan genotype with an index of 15.09 was able to have the highest seedling length vigor index under the treatment of combined foliar application of nano zinc oxide and zinc sulfate under normal irrigation conditions. Also, comparison of the mean data showed that the combined use of foliar spraying of nano zinc oxide and zinc sulfate was able to moderate the effect of drought stress on the seedling length vigor index, so that the seedling length vigor index of the Zafar genotype was 15.72 under mild drought stress conditions (water cut-off at the pod formation stage) and 13.29 under severe drought stress conditions (water cut-off at the flowering stage), which was superior to other cultivars and treatments (Figure 9).

**Table 6:** Duncan’s Mean Comparison Test: Effect of Year on Traits GE, SLVI, PL, RL and AC.

**Year**	**GE**	**SLVI**	**PL**	**RL**	**AC**
first year (2021)	96.8152 a	13.3661 a	5.81875 a	7.78333 a	0.748156 a
second year (2022)	96.625 a	12.9038 b	5.70625 a	7.4625 b	0.765445 a

Numbers that have at least one letter in common in each column have no significant difference with Duncan’s test at the 5% level. GE is Germination Energy (-); SLVI is Seedling length Vigor Index (-); PL is Plumule length (cm); RL is Root length (cm); AC is Allometric Coefficient (-).

### Plumule length (PL)

The results of the variance analysis showed that the effects of irrigation levels, zinc fertilizer levels, and genotype on plumule length were significant at the 1% probability level, and the interaction effect of irrigation levels by zinc fertilizer levels was significant at the 5% probability level (Table 5.).

**Fig 10:**
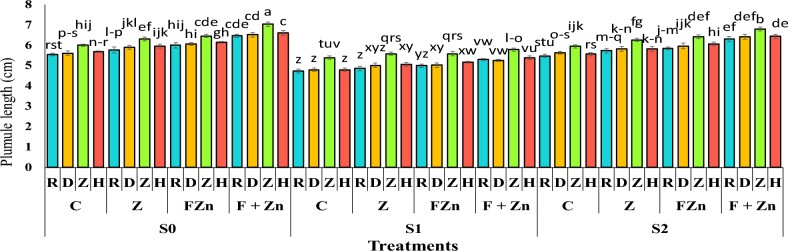
The combined effect of treatments on plumule length in two years of experiment. Stress levels: S0 – No stress; S1 - Stress in flowering; S2 - Stress in pod formation. Zinc fertilizer levels: C – Control; Z - Zinc sulfate; FZN - Foliar zinc Nano oxide; F+ZN - Foliar zinc Nano oxide + Zinc sulfate. Canola genotypes: R – RGS003; D – Delgan; Z – Zafar; H- Hyola 50. Bars that have at least one letter in common are not significantly different by Duncan’s test at the 5% probability level.

The results of the mean comparison showed that the most excellent plumule length, with an average of 7.01 cm, was associated with the seeds of the open-pollinated Zafar genotype in the treatment of combined foliar application of nano zinc oxide and zinc sulfate, under normal irrigation conditions (without irrigation stress). After that, the hybrid genotype Hyola50 had a greater plumule length than other cultivars under the same conditions, averaging 6.58 cm. Also, the comparison of the mean data showed that the combined use of foliar spraying of nano zinc oxide and zinc sulfate was able to modulate the effect of drought stress on plumule length to a greater extent than other zinc treatments, so that the plumule length of the Zafar genotype under mild drought stress conditions (water cut-off at the pod formation stage) was 6.78 cm and under severe drought stress conditions (water cut-off at the flowering stage) was 5.76 cm, which was superior to other cultivars and treatments (Figure 10).

### Root length (RL)

The results of the variance analysis showed that year, irrigation level, zinc fertilizer level, and genotype had significant effects on root length at the 1% level of significance (Table 5.).

**Fig 11:**
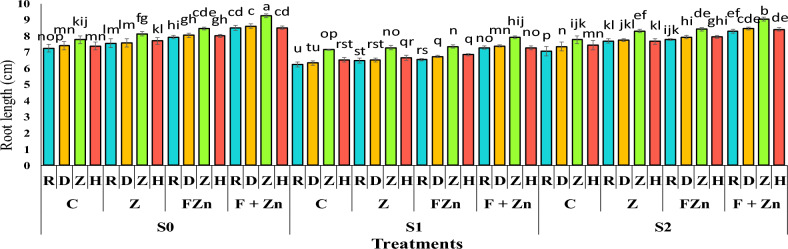
The combined effect of treatments on root length in two years of experiment. Stress levels: S0 – No stress; S1 - Stress in flowering; S2 - Stress in pod formation. Zinc fertilizer levels: C – Control; Z - Zinc sulfate; FZN - Foliar zinc Nano oxide; F+ZN - Foliar zinc Nano oxide + Zinc sulfate. Canola genotypes: R – RGS003; D – Delgan; Z – Zafar; H- Hyola 50. Bars that have at least one letter in common are not significantly different by Duncan’s test at the 5% probability level.

The results of mean comparison showed that the root length in the first year was 7.78 cm longer than in the second year with an average of 7.46 (Table 6.). the most extended root length was related to the seeds of the open-pollinated Zafar genotype with an average length of 9.25 cm in the treatment of combined foliar application of nano zinc oxide and zinc sulfate under normal irrigation conditions (without irrigation stress). After that, the Delgan genotype had a longer root length than other cultivars under the same conditions, averaging 8.61 cm. Also, the comparison of the mean data showed that the combined use of foliar spraying of nano zinc oxide and zinc sulfate was able to modulate the effect of drought stress on root length to a greater extent than other zinc treatments, so that the root length of the Zafar genotype under mild drought stress conditions (water cut-off at the pod formation stage) was 9.05 cm and under severe drought stress conditions (water cut-off at the flowering stage) was 7.91 cm, which was superior to other cultivars and treatments (Figure 11).

### Allometric coefficient (AC)

The results of variance analysis showed that the interaction effect of year on irrigation levels, the interaction effect of year on zinc fertilizer levels, and the combined effect of year on irrigation levels on zinc fertilizer levels on the allometric coefficient were significant at the 1% probability level. (Table 5.).

**Fig 12:**
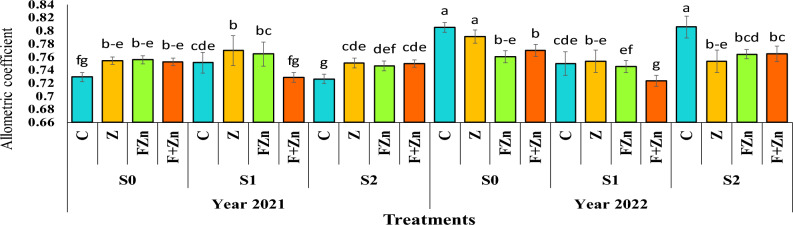
The reciprocal effect of year on irrigation levels on Allometric coefficient. Stress levels: S0 – No stress; S1 - Stress in flowering; S2 - Stress in pod formation; C – Control; Z - Zinc sulfate; FZN - Foliar zinc Nano oxide; F+ZN - Foliar zinc Nano oxide + Zinc sulfate. Bars that have at least one letter in common are not significantly different by Duncan’s test at the 5% probability level.

The results of comparing the mean data showed that the highest allometric coefficient in the second year with a mean of 0.805 was related to seeds obtained from the mother plant under conditions of no irrigation stress and mild irrigation stress (water cut-off at the pod formation stage) in the treatment without the use of zinc fertilizer, both of which were placed in statistical group a, and the lowest allometric coefficient with a mean of 0.723 was related to seeds obtained from the mother plant under conditions of severe water stress (water cut-off at the flowering stage) and the fertilizer treatment with the combined use of zinc sulfate and nano zinc oxide (Figure 12).

### Year effect

A comparison of the mean data revealed that traits such as GP, MDG, GV, SLVI, and RL were more significant in 2021, the first year of the experiment, than in 2022, the second year (Tables 4. and 6.).

### Correlations

Correlation analysis indicated that GP was positively correlated with the MDG, GV, SLVI, PL, and RL indices and negatively correlated with GR and GE. The allometric coefficient (AC) was positively correlated with the mean germination time (MGT) and negatively correlated with the germination energy (GE) and germination rate (GR). Negative correlations were found between GR and the MDG, GV, SLVI, AC, PL, and RL indices. MDG was positively correlated with the GV, SLVI, PL, and RL indices but negatively correlated with GE. There were positive correlations between the germination value (GV) and the SLVI, PL, and RL indices, but negative correlations with the GE index. The correlations of GE with the SLVI, AC, PL, and RL indices were negative. The PL and RL indices were correlated with the SLVI, and the PL showed a positive correlation with the RL (Table 7.).

**Table 7:** Treatments correlations.

Treatments	GP	MGT	GR	MDG	GV	GE	SLVI	PL	RL	AC
GP	1									
MGT	0.188	1								
GR	−0.047	−0.102	1							
MDG	0.915^**^	0.189	−0.047	1						
GV	0.954^**^	0.187	−0.047	1.000^**^	1					
GE	−0.219	−0.918	0.181	−0.221	−0.217	1				
SLVI	0.758^**^	0.114	−0.093	0.758^**^	0.757^**^	−0.143	1			
PL	0.733^**^	0.147	−0.105	0.733^**^	0.732^**^	−0.183	0.995^**^	1		
RL	0.712^**^	0.071	−0.085	0.712^**^	0.711^**^	−0.096	0.995^**^	0.986^**^	1	
AC	0.276	0.520^**^	−0.151	0.279	0.275	−0.571	0.185	0.266	0.103	1

ns, *, **: Representing nonsignificant and significant effects at 5 and 1% probability levels.

## Discussion

In recent years, drought stress has been recognized as a significant environmental factor that severely impacts the productivity of many crops, delaying their growth and development^33^. Gutterman (2000) reported that seed germination can be influenced by maternal factors, such as the seed’s position on the plant, the age of the mother plant during seed maturation, and environmental factors, including day length, temperature, light quality, water availability, and altitude^34^. Studies have shown that under non-stress conditions, there is no significant difference in germination percentage among cultivars, whereas under stress conditions, substantial differences are observed. The Hyola401 and Modena cultivars exhibited the lowest germination percentages, with 94% and 93% reduction in germination indices, respectively, thereby identifying them as the most sensitive cultivars studied. Conversely, the Licord cultivar, followed by the Okapi cultivar, presented the least reduction in germination under severe stress conditions. Therefore, these cultivars exhibit high drought tolerance at the germination stage, and, in general, cultivars showing less variation across environments demonstrate greater drought tolerance during germination^5^. Applying drought stress to the mother plant reduced the percentage of germinated seeds, as drought stress directly and indirectly affects seed metabolism, leading to seed shrinkage and reduced nutrient reserves^35^. Under end-of-season drought conditions, photosynthesis rates rapidly decrease^36^. Thus, insufficient material transfer occurs during seed filling^37^. When drought stress happens, and seed filling relies heavily on remobilizing materials from the stem to the seed, this transfer may be insufficient, resulting in reduced seed weight^38^. This reduction in seed weight, or, in other words, decreased reserves, results in a lower germination percentage for the obtained seeds. Seeds that can maintain acceptable germination under stress conditions are highly valuable in arid and semiarid regions, as they are more likely to survive during this critical stage^39^. Higher maternal seed reserves lead to greater plumule and root growth, resulting in more vigorous, healthier seedlings. By reducing maternal seed reserves and seed quality, drought stress decreases the seed vigor index, resulting in weaker plumules and root growth, and ultimately weaker seedlings^40^.

In the present study, results showed that applying drought stress to the mother plant decreased the germination percentage of the produced seeds, as drought stress directly and indirectly affects seed metabolism, leading to shrinkage and reduced seed reserves. In other words, in dry conditions at the end of the season, the rate of photosynthesis decreases rapidly, and thus, the transfer of materials to fill the seed will not be sufficient. When drought stress occurs, and seed filling requires a strong transfer of photosynthetic materials from the stem to the seed, this transfer is insufficient, resulting in decreased seed weight. This decrease in seed weight, or in other words, a decrease in its reserves, will lead to a decrease in the germination percentage of the obtained seeds. Certainly, seeds that can germinate under acceptable stress conditions will be of great value in arid and semi-arid regions, as they are more likely to pass this stage. In our experiment, we found that the combined use of foliar applications of nano zinc oxide and zinc sulfate partially reduced the effect of drought stress on germination percentage. It is essential to adjust the seeds, and the more reserves the mother seeds have, the higher the growth rates of the stem and root, resulting in stronger, healthier seedlings. Drought stress, which reduces maternal seed reserves and seed quality, decreases the seed vigor index and reduces shoot and root growth, ultimately leading to the production of weaker seedlings. As stress on the mother plant increased, seed vigor index, root length, and shoot length decreased. Since the effect of drought stress on the pollination and seed filling stages, on the one hand, caused the loss of pollen grains and, on the other hand, disrupted the production and transfer of photosynthetic materials to the seed, it ultimately resulted in a decrease in seed weight. The decrease in germination indices, such as germination percentage, root length, shoot length, and seed vigor index, observed in this experiment, appears to be attributed to a slight decline in seed weight and quality. The reason that germination rate (GR) and two traits, mean germination time (MGT) and germination energy (GE), which are directly related to germination rate, were not affected by drought stress may be associated with the genetic structure of the plant or that the difference in seed size is to some extent It should not make a difference in the speed of germination.

Chlorophyll synthesis, pollen function, and germination require zinc^22^. The results indicated that drought stress, nutrient application, and foliar application timing significantly affected germination percentage (GP), speed (GS), value (GV), and energy (GE) at the 1% level. Other researchers have also reported that foliar nutrient application can reduce the effects of stress on seed vigor^41^. Studies have shown that drought stress, nutrient application, and foliar spray time have a significant impact at the one percent level on germination percentage (GP), germination rate (GR), germination value (GV), and germination energy (GE). Main effects analysis revealed that the maximum GP, GR, GV, and GE were associated with control conditions (without drought stress), drought stress at 50% flowering, and treatment with nano-zinc and nano-iron nutrients^12^. Studies have shown that drought stress and micronutrient application to the mother plant effectively elicited distinct responses in germination-related traits. Nanomaterials have recently been widely used in various scientific fields, including pharmaceutical, medicinal, physical, and agricultural sciences^42^. These materials have been used in several farming practices due to their efficacy in plant protection and nutrition^43^. Zinc plays a crucial role in various physiological and biochemical processes within plant cells, including protein synthesis, membrane function, and gene expression. This metal component acts as a regulatory element for many enzymes and plays a significant role in plant defense systems against stress conditions^44,45^.

In the present study, the use of zinc micronutrients in the form of sulfate on the soil and the foliar application of nano zinc oxide on the mother plant under drought stress conditions significantly increased all germination indices except MGT and AC. This could be because zinc affects growth and cell division through the production of growth hormones, such as auxin, which stimulates root growth and leaf development. The lack or deficiency of zinc causes leaves to shrink, root growth to decrease, and results in plant dwarfism. This element also plays a role in increasing photosynthesis and energy production, as well as maintaining the stability of the cell membrane and chloroplast structure. Enhancing the activity of enzymes involved in photosynthesis increases dry matter production and promotes better rapeseed growth. On the other hand, it enhances stress tolerance by regulating antioxidants, such as superoxide dismutase, which help reduce the destructive effects of environmental stresses, including drought. Ultimately, increased flower formation and better fertilization result in seeds with greater weight and quality. It will be higher, thereby positively affecting seed germination indices. Overall, it can be concluded that the use of zinc micronutrients, whether through soil application or foliar spraying with nano-zinc oxide, can enhance seedling drought tolerance. However, the combination of foliar spraying with nano-zinc oxide yielded the best results.

The germination time course of 10 rapeseed seeds was investigated in an experiment, in which the Okapi genotype had the highest germination percentage (89%) after 1 day, and the Hyola 330 genotype had 0%. However, along with the Zerfam genotype (99%), the highest final germination percentages were observed for RGS003, Elite, Option 500, Slmo 46, and Hyola 401 (95–97%). The main objective of this experiment was to investigate the average germination time MGT for evaluating 10 rapeseed seed varieties. Seeds that germinate more slowly in our study produce a higher number of abnormal seedlings, and MGT can be used as an indicator of seed vigor in rapeseed^13^. Studies have shown that seed vigor tests significantly affect abnormal seedling weight, height, mean germination time (MGT), daily germination rate (DGR), mean daily germination rate (MDGR), and final germination percentage (FGP). Laboratory-measured data showed that the standard germination test had a positive effect on root-to-shoot ratio, MGT, seedling fresh and dry weight, full seedling height, shoot height, and root length^46^.

In the present experiment, the studies showed that the seeds of the hybrid genotype Hyola 50 were superior to other genotypes in GP, ​​MDG, and GV indices, but did not differ from other genotypes in other germination indices. The open-pollinated genotype Zafar was also superior to other genotypes in SLVI, PL, and RL indices. With a high seed vigor index and longer root and shoot lengths, this genotype can establish itself more successfully in the early stages, a highly desirable characteristic under drought stress. Seeds from the cultivars in the first year of the experiment were superior to those in the second year in terms of the most critical indicators studied, namely germination percentage (GP) and seed vigor index (SVI). It appears this was due to better plant utilization resulting from more favorable weather conditions in the first year of the experiment. Overall, by examining the three-dimensional effects of zinc treatments on cultivar germination under water stress, it can be concluded that the combined use of zinc sulfate and foliar spraying with nano zinc oxide had a positive effect on the germination indices of seeds from cultivars under both water-stress and non-water-stress conditions. Still, the superiority of the genotypes in some index seemed to be related to the cultivar’s genetic characteristics. However, in general, the open-pollinated Delgan genotype, following the hybrid Hyola 50, had better germination indices than the other two cultivars.

Studies have shown that cultivars with high germination capacity do not necessarily have good seedling growth^5^. However, in the present experiment, using trait correlations, it was determined that cultivars with a high germination percentage (GP) were superior to others in terms of other germination indices. Additionally, the trait correlation analysis showed that cultivars with higher seed vigor indices had longer root and shoot lengths. Among them, the Zafar genotype, when combined with the use of foliar nano zinc oxide and zinc sulfate under normal irrigation conditions, was superior to other cultivars. However, a genotype superior in these traits may not necessarily be selected as superior under normal irrigation conditions. Still, it can be considered a good option for planting under water-stress conditions.

## Conclusion

Overall, this study demonstrated that drought stress conditions, from the onset of flowering to the grain-filling period, inhibited all germination indices of productive rapeseed. The use of zinc fertilizer in the form of combined application of sulfate on the soil and foliar application of nano zinc oxide at a concentration of 5 5ppm in the three growth periods of seedling, flowering, and pod formation on the mother plant significantly increased the germination indices of the obtained seeds. Meanwhile, the seeds of the hybrid genotype Hyola 50, under the same treatment and in both standard and water-stress conditions, showed slightly higher germination indices than those of the Delgan genotype. However, they were more superior to the two genotypes, RGS003 and Zafar, by a greater margin. It can be concluded that, in the absence of access to hybrid seeds, farmers in the region can use the open-pollinated Delgan genotype, which is easier to reproduce and has germination characteristics similar to those of the hybrid Hyola 50 under both normal and water-stress conditions. However, further complementary field experiments over several years are needed to confirm that the studied treatments improve rapeseed seed germination indices and enhance resistance to drought stress in the field.

## Data Availability

All data supporting the results are included in this article, and no additional source data are required.
